# Declining rural oncologist supply in the United States: a workforce analysis from 2014 to 2025

**DOI:** 10.1093/oncolo/oyag333

**Published:** 2026-08-19

**Authors:** David X Zheng

**Affiliations:** Mass General Brigham Cancer Institute, Massachusetts General Hospital, Harvard Medical School, Boston, MA 02114, United States; Department of Medicine, Massachusetts General Hospital, Harvard Medical School, Boston, MA 02114, United States; Broad Institute of MIT and Harvard, Cambridge, MA 02142, United States

**Keywords:** rural health, oncology workforce, health disparities, rural–urban commuting area, cancer care access

## Abstract

**Purpose:**

Rural Americans experience higher cancer incidence and mortality than their urban counterparts, yet oncology physician supply to rural areas is incompletely characterized. We determined longitudinal trends in the proportion of oncologists practicing rurally and identified characteristics associated with rural practice.

**Methods:**

Physicians reporting a primary specialty of hematology, hematology/oncology, or medical oncology were identified from annual archived versions of the CMS Physician Compare Database (2014-2025). Rural status was determined from Rural-Urban Commuting Area codes applied to practice ZIP code (codes 4-10 = rural). Temporal trends were assessed with Mann-Kendall tests. Rural and urban physician characteristics were compared using chi-squared tests and multivariable logistic regression.

**Results:**

We identified 19,807 unique oncologists (14,272 in 2025). Despite a 26% workforce expansion, the rural proportion declined from 11.1% to 8.0% (τ = −0.73, *P *= .001). Per capita, rural supply fell from 6.5 to 5.5 per 100,000 adults aged ≥55 (τ = −0.58, *P *= .009) while urban supply rose from 11.7 to 13.1 (τ = +0.48), widening the urban-to-rural ratio from 1.8 to 2.4. Rural oncologists were more likely to be male, late career, and in mid-size groups (all *P *< .001). Academic medical center affiliation (aOR 0.10) and large group size (aOR 0.40) were associated with decreased odds of rural practice, whereas late career stage (aOR 1.58) and Midwest region (aOR 1.62) were associated with increased odds.

**Conclusions:**

The rural oncology workforce is declining proportionally and per capita despite overall growth. Retirement of late-career rural oncologists without early-career replacement threatens to further erode rural cancer care access.

Implications for practice The proportion of oncologists practicing in rural areas declined in both relative and per capita terms from 2014 to 2025 despite overall workforce growth, with rural practice disproportionately sustained by late-career physicians unlikely to be replaced by early-career oncologists. These findings suggest that, without deliberate intervention, rural cancer care access will continue to erode. Clinicians, training programs, and policymakers should prioritize rural fellowship exposure, targeted loan repayment for rural oncology practice, and policies sustaining community oncology infrastructure—the nearly exclusive vehicle through which rural Americans access cancer care.

## Introduction

Rural Americans bear a disproportionate burden of cancer, including higher overall incidence,^1^ stage at diagnosis,^2^ and mortality.^3^ Geographic access to oncology care is among the contributors most consistently linked to these disparities, with rural patients often facing transportation barriers to healthcare,^4^ reduced clinical trial enrollment,^5^ and delays from symptom onset to diagnosis/treatment initiation.^6^ Although geographic access is consistently associated with these outcomes, it coexists with and therefore is challenging to disentangle from socioeconomic disadvantage, lower health literacy, and insurance-related barriers that independently shape rural cancer outcomes.^7^

The distribution of the oncology physician workforce is a central determinant of rural cancer care access. Given the above, physician supply is best understood as one modifiable component of a multifactorial access problem rather than as an isolated cause of the disproportionate cancer burden faced by rural Americans. Prior analyses have revealed a substantial urban concentration of oncologists in the United States, with rural areas relying disproportionately on a small, potentially aging cohort of community-based physicians.^8–10^ National projections anticipate a widening gap between oncologist supply and demand over the coming decade, driven by an aging population, expanding treatment indications, and increasing treatment complexity, pressures likely to fall most heavily on already underserved rural communities.^11–13^

Despite these trends, a comprehensive, longitudinal characterization of the rural oncology workforce is lacking. Prior studies have largely been limited by cross-sectional design,^14^ focus on patient-level access metrics rather than physician supply,^15^ or inclusion of oncology within broader specialist shortage analyses without isolation of rural trends over time.^16^ Using eleven years of nationally comprehensive physician enrollment data, we sought to characterize temporal trends in the proportion of oncologists practicing in rural areas and to identify physician and practice characteristics independently associated with rural practice location.

## Methods

### Data source and study population

Annual archived versions of the CMS Physician Compare Database were obtained for each year from 2014 through 2025. This publicly available dataset is derived from the Provider Enrollment, Chain, and Ownership System (PECOS) and contains self-reported professional and practice information for physicians servicing Medicare patients. We extracted all physicians self-reporting a primary specialty of hematology, hematology/oncology, or medical oncology–3 distinct PECOS designations that collectively encompass the physician workforce responsible for diagnosing and treating malignant solid tumors and hematologic disease in the United States. As the majority of fellowship-trained oncology physicians pursue combined hematology/oncology board certification through the American Board of Internal Medicine,^17^ a single physician may appear under more than one specialty designation across data files within a given year. Records were therefore deduplicated using the National Provider Identifier as a unique physician key; when duplicates existed within a year, the self-reported primary specialty field determined the retained record. The combined dataset was treated as a repeated cross-sectional series of annual cohorts spanning 2014-2025. For brevity, physicians within these 3 designations are referred to collectively as “oncologists” throughout the manuscript.

### Practice location and rural classification

Practice ZIP codes were linked to the 2010 vintage ZIP code Rural-Urban Commuting Area (RUCA) code file published by the USDA Economic Research Service. RUCA codes classify ZIP codes along a 1-10 continuum using population density and commuting flow data derived from decennial census and American Community Survey estimates. We defined rural practice as a RUCA code of 4-10 and urban practice as a RUCA code of 1-3, consistent with established conventions in rural health research.^18–20^ Physicians with multiple practice locations spanning both classifications were assigned rural status. To account for the size of the at-risk population, we additionally calculated oncologist supply per 100,000 adults aged 55 years or older, separately for rural and urban areas, using age-specific denominators from the American Community Survey 5-year estimates at the ZIP code tabulation area level classified with the same RUCA crosswalk. In sensitivity analyses, the annual rural population was recomputed under a stricter definition limited to small town and isolated areas (RUCA 7-10) and with exclusion of ZIP codes assigned to metropolitan areas via secondary commuting flows.

### Covariate definitions

Covariates were selected *a priori* based on their availability within the dataset and theoretical relevance to geographic patterns. Sex was obtained directly from PECOS enrollment records. Career stage was derived from medical school graduation year, subtracting graduation year from the study year and applying a 6-year offset to approximate post-graduate training duration, providing an estimated years-in-practice value subsequently grouped as early (0-9 years), mid (10-24 years) or late (≥25 years). Practice group size was stratified by small (1-9), medium (10-99), or large (≥100); the group size field in the dataset reflects the size of the entire Medicare-enrolled billing organization rather than the local practice site. US Census region was determined based on state of practice.

Academic medical center (AMC) affiliation required satisfying 2 conditions simultaneously: (1) the physician’s practice city and state corresponded to a location with an ACGME-accredited hematology/oncology fellowship program whose sponsoring institution is affiliated with an LCME-accredited MD-granting medical school and (2) the enrolled organization name contained a keyword (eg, “university,” “school of medicine”) consistent with AMC designation. This definition was chosen to anchor AMC classification to the dual institutional functions most directly relevant to oncology workforce production (ie, structured graduate medical training and formal medical school affiliation) rather than relying on organization name alone.

Specialty was retained as a 3-level variable (hematology, hematology/oncology, medical oncology) reflecting the self-reported primary specialty in PECOS. Given that hematology/oncology represents the predominant training and certification pathway, it served as the reference category in regression analyses.

### Statistical analysis

Descriptive statistics were generated for the 2025 cross-section, as this was the most recent available year. Continuous variables are reported as proportions with 95% Wilson score confidence intervals. Differences in physician and practice characteristics between rural and urban cohorts were evaluated using chi-squared tests. Annual rural proportions were calculated across all twelve study years, and monotonic temporal trends were assessed using Mann–Kendall test, a non-parametric method for detecting trends in time-series data without assuming linearity.^21^ Separate trend tests were applied to annual rural proportions, total cohort count, and absolute rural physician count.

Using the 2025 cross-section, we fit a multivariable logistic regression model with rural versus urban practice as the binary outcome. All covariates were inputted simultaneously; no stepwise or automated variable selection was performed as this approach has been shown to introduce bias in observational settings.^22^ Model discrimination was quantified by area under the receiver operating characteristic curve (AUC). Variance inflation factors (VIFs) were calculated for all predictors, with values exceeding 5.0 considered to indicate substantial collinearity. Physicians with missing values on any modeled covariate were excluded from the regression (ie, complete case analysis); overall missingness across covariates was less than 3%. All analyses were completed in R version 4.4.3. All hypothesis tests were 2-sided; statistical significance was defined as *P *< .05.

## Results

### Study population and temporal trends

**Figure 1.**
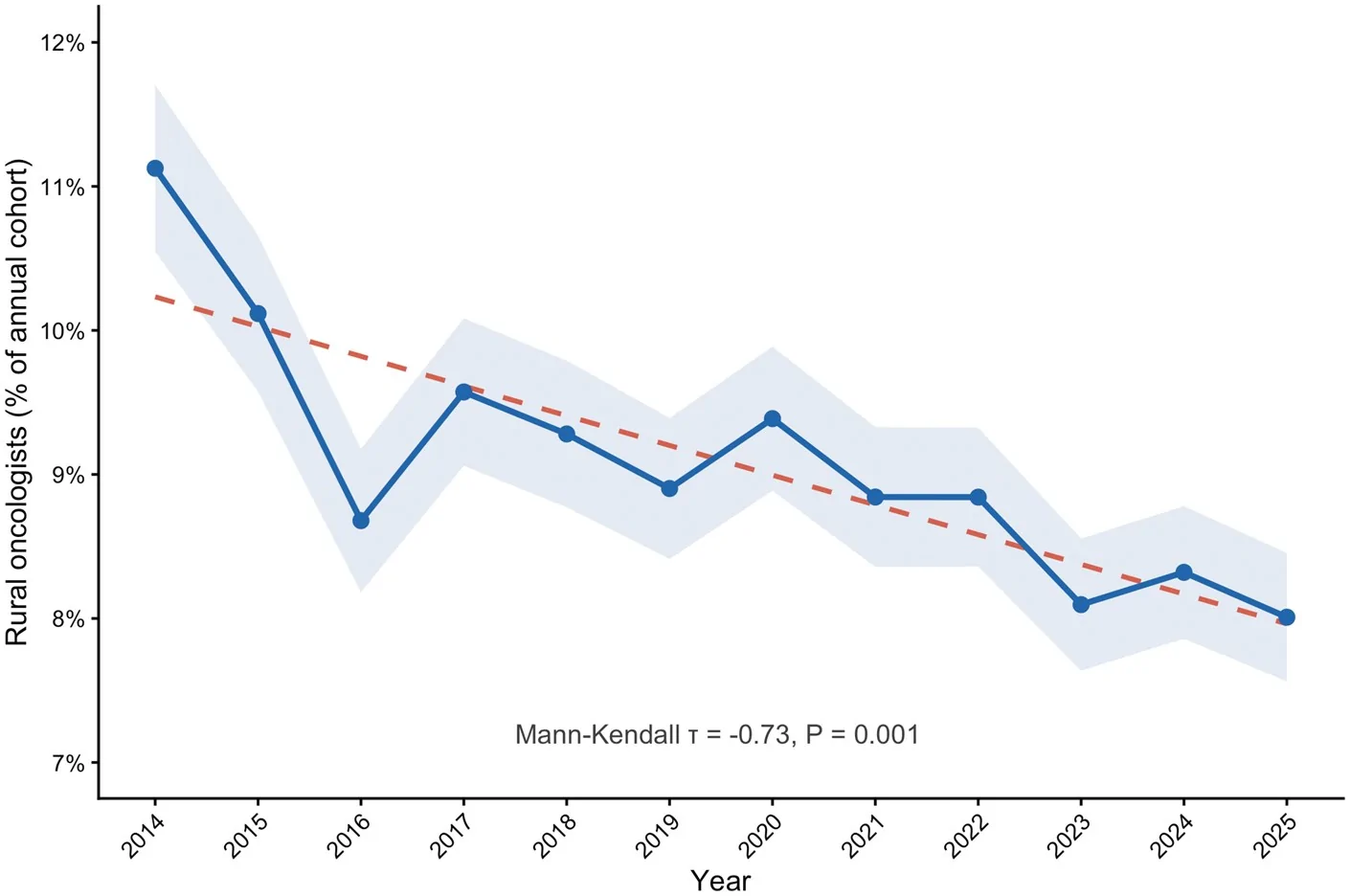
Trend in US rural oncology physician representation, 2014-2025. Proportion of oncology physicians (hematology, hematology/oncology, medical oncology) practicing in rural areas (RUCA codes 4-10) per year in the CMS Physician Compare database. Shaded band indicates 95% Wilson confidence interval. Dashed red line indicates ordinary least squares trend. Mann–Kendall τ = −0.73, *P* = .001 (decreasing trend).

**Figure 2.**
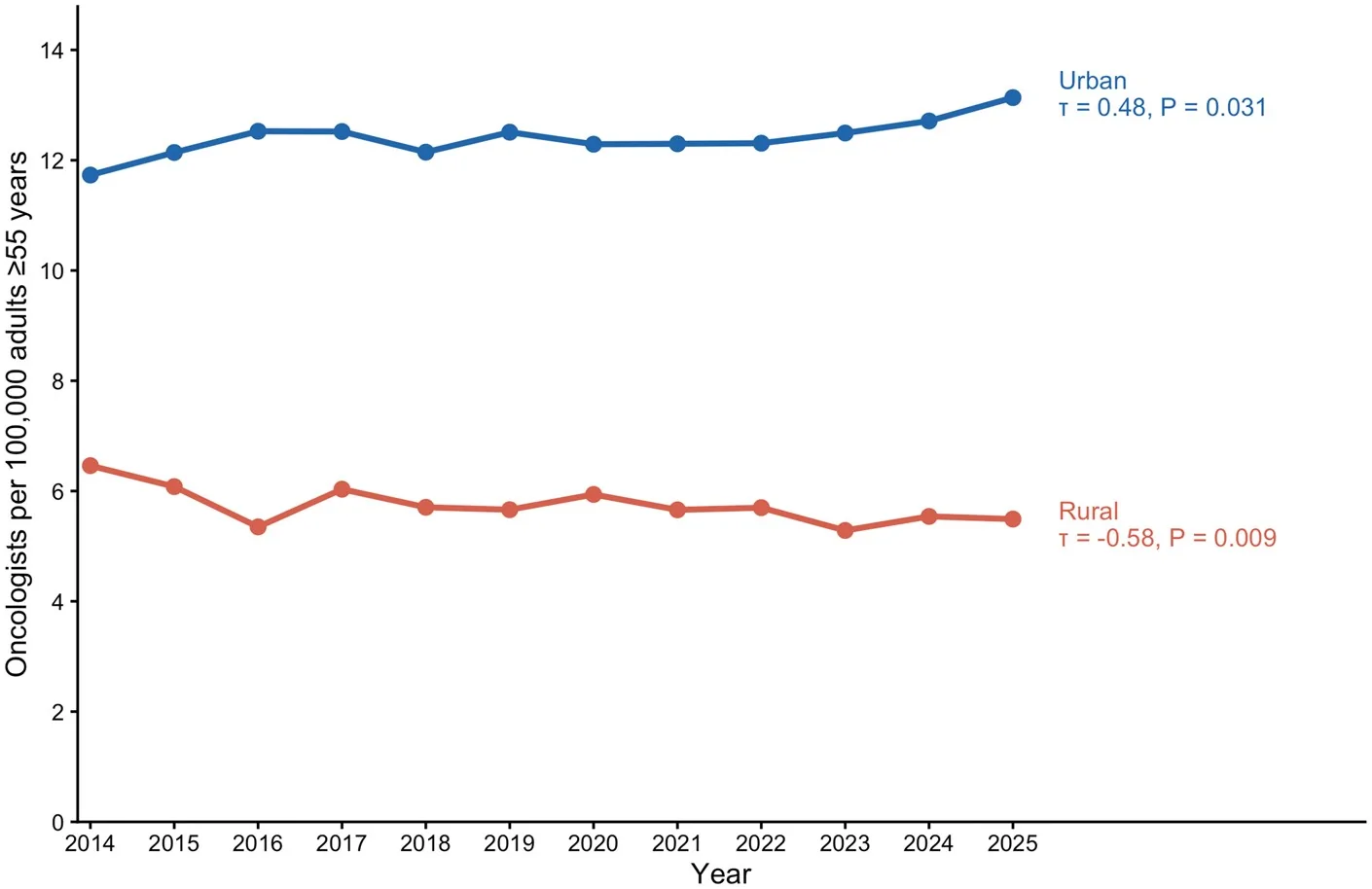
Oncologist supply per 100,000 adults aged ≥55 years, rural versus urban, 2014-2025. Numerators are the annual rural and urban oncologist counts. Denominators are the population aged ≥55 years from American Community Survey 5-year estimates at the ZIP code tabulation area level, classified as rural or urban with the same RUCA crosswalk. Mann–Kendall rural τ = -0.58, *P* = .009; urban τ = +0.48, *P* = .03.

Across all twelve annual cross-sections, 19,807 unique oncologists were identified. The active workforce grew steadily throughout the study period, increasing from 11,352 physicians in 2014 to 14,272 in 2025, a 26% expansion (*P *< .001). The absolute number of oncologists practicing in rural settings fluctuated without a statistically significant directional trend over the study period (1263 in 2014 vs 1143 in 2025, *P *= .12). As a consequence of urban-concentrated growth, the rural proportion of the oncology workforce declined significantly, from 11.1% (95% CI 10.5-11.7) in 2014 to 8.0% (95% CI 7.6-8.5) in 2025 (Mann–Kendall τ = −0.73, *P *= .001; Figure 1). When stratified by the population aged 55 years or older, rural oncologist supply fell from 6.5 to 5.5 per 100,000 (τ = −0.58, *P *= .009), while urban supply rose from 11.7 to 13.1 per 100,000 (τ = +0.48, *P *= .03). The urban-to-rural density ratio consequently widened from 1.8 to 2.4 (τ = +0.58, *P *= .009; Figure 2).

### Physician and practice characteristics

Among the 14,272 oncologists active in 2025, 1143 (8.0%) practiced in rural settings. Hematology/oncology was the predominant specialty designation overall (66.5%), followed by medical oncology (26.7%) and hematology (6.8%). Rural and urban oncologists differed significantly across every measured characteristic (Table 1; all *P* < .001). Compared to their urban counterparts, the rural workforce was more male predominant (70.2% vs 61.7%) and skewed toward later career stages: 39.8% of rural oncologists (compared to 29.1% of urban) were late career (≥25 years of practice), while early-career oncologists (0-9 years) were underrepresented rurally (18.8% vs 27.6%). AMC affiliation was nearly absent in rural practice, as only 2.3% of rural oncologists practiced at confirmed AMCs compared to 24.2% of urban oncologists. Geographically, the Midwest accounted for nearly one-third of rural oncologists (32.7%) despite representing only 20.2% of the urban oncology workforce; the Northeast and West were each underrepresented in rural relative to urban settings. Within specialty, hematologists were modestly less represented rurally (2.6% vs 7.1%), while the distribution of hematology/oncology and medical oncology was more balanced between settings. Regarding group size, rural oncologists more commonly practiced in medium-sized practices (10-99; 36.1% vs 14.9%), whereas large groups (≥100) were modestly more common in urban settings (58.8% vs 81.4%). Median group size in 2025 was 537 (interquartile range 121-1681).

**Table 1.** Characteristics of US oncology physicians in 2025.

	Overall (*N* = 14,272)	Rural (*N* = 1143)	Urban (*N* = 13,129)	*P*-value
**Rural location**	8.0% (7.6-8.5)	—	—	
**Sex**				
** Male**	62.4%	70.2%	61.7%	<.001
** Female**	37.6%	29.8%	38.3%	
**Career stage (years since graduation)**				
** Early career (0-9 yr)**	26.9%	18.8%	27.6%	<.001
** Mid-career (10-24 yr)**	43.1%	41.4%	43.3%	
** Late career (≥25 yr)**	30.0%	39.8%	29.1%	
**Academic affiliation**				
** AMC**	22.4%	2.3%	24.2%	<.001
** Non-AMC**	77.6%	97.7%	75.8%	
**Practice group size**				
** Solo/small (1-9)**	3.8%	5.1%	3.7%	<.001
** Medium (10-99)**	16.6%	36.1%	14.9%	
** Large (≥100)**	79.6%	58.8%	81.4%	
**Census region**				
** South**	33.9%	36.9%	33.7%	<.001
** Midwest**	21.2%	32.7%	20.2%	
** Northeast**	24.0%	14.7%	24.8%	
** West**	20.8%	14.6%	21.4%	
**Specialty**				
** Hematology/oncology**	66.5%	70.5%	66.2%	<.001
** Medical oncology**	26.7%	26.8%	26.7%	
** Hematology**	6.8%	2.6%	7.1%	

AMC = academic medical center (practice city and state hosts ACGME hematology/oncology fellowship affiliated with LCME-accredited medical school, and organization name contains academic medical center keyword), RUCA = rural–urban commuting area.

Physicians are classified by RUCA code applied to practice ZIP code (rural: codes 4-10; urban: codes 1-3; 2010 USDA vintage). All comparisons between rural and urban groups: *P *< .001 by chi-squared test.

### Factors associated with rural practice

The multivariable logistic regression model demonstrated good discrimination (AUC = 0.74), and no predictor exhibited concerning collinearity (all VIFs <2.0). Results are presented in Table 2. AMC affiliation (aOR 0.10, 95% CI 0.07-0.15), large (aOR 0.40, 95% CI 0.35-0.46) and small (aOR 0.57, 95% CI 0.42-0.77) group size, female sex (aOR 0.86, 95% CI 0.75-0.99), and practice in the Northeast (aOR 0.61, 95% CI 0.50-0.74) or West (aOR 0.63, 95% CI 0.52-0.76) relative to South were associated with lower odds of rural practice. Late- (aOR 1.58, 95% CI 1.32-1.88) and mid- (aOR 1.21, 95% CI 1.02-1.44) career stage, as well as practice in the Midwest (aOR 1.62, 95% CI 1.39-1.88), were associated with higher odds of rural practice. Within specialty, relative to hematology/oncology, hematology designation (aOR 0.55, 95% CI 0.38-0.81) was associated with lower odds of rural practice and medical oncology designation (aOR 1.20, 95% CI 1.04-1.39) was associated with higher odds of rural practice.

**Table 2.** Logistic regression of rural practice location among US oncology physicians, 2025.

Variable	aOR	95% CI	*P*-value
**Sex**			
** Female**	0.86	0.75-0.99	.037
**Career stage**			
** Mid-career (10-24 yr)**	1.21	1.02-1.44	.030
** Late career (≥25 yr)**	1.58	1.32-1.88	<.001
**Academic affiliation**			
** Academic medical center**	0.10	0.07-0.15	<.001
**Practice group size**			
** Solo/small (1-9)**	0.57	0.42-0.77	<.001
** Large (≥100)**	0.40	0.35-0.46	<.001
**Census region**			
** Midwest**	1.62	1.39-1.88	<.001
** Northeast**	0.61	0.50-0.74	<.001
** West**	0.63	0.52-0.76	<.001
**Specialty**			
** Medical oncology**	1.20	1.04-1.39	.010
** Hematology**	0.55	0.38-0.81	.002

aOR = adjusted odds ratio; AUC = area under the receiver operating characteristic curve; VIF = variance inflation factor.

Outcome: rural vs urban practice location. All variables entered simultaneously (no stepwise selection). Complete case analysis (*N* = 13,891). AUC = 0.741; all VIF < 2.0. Reference categories: male sex, early career (0-9 yr), non-AMC, medium group size (10-99), South region, hematology/oncology specialty.

**Figure 3.**
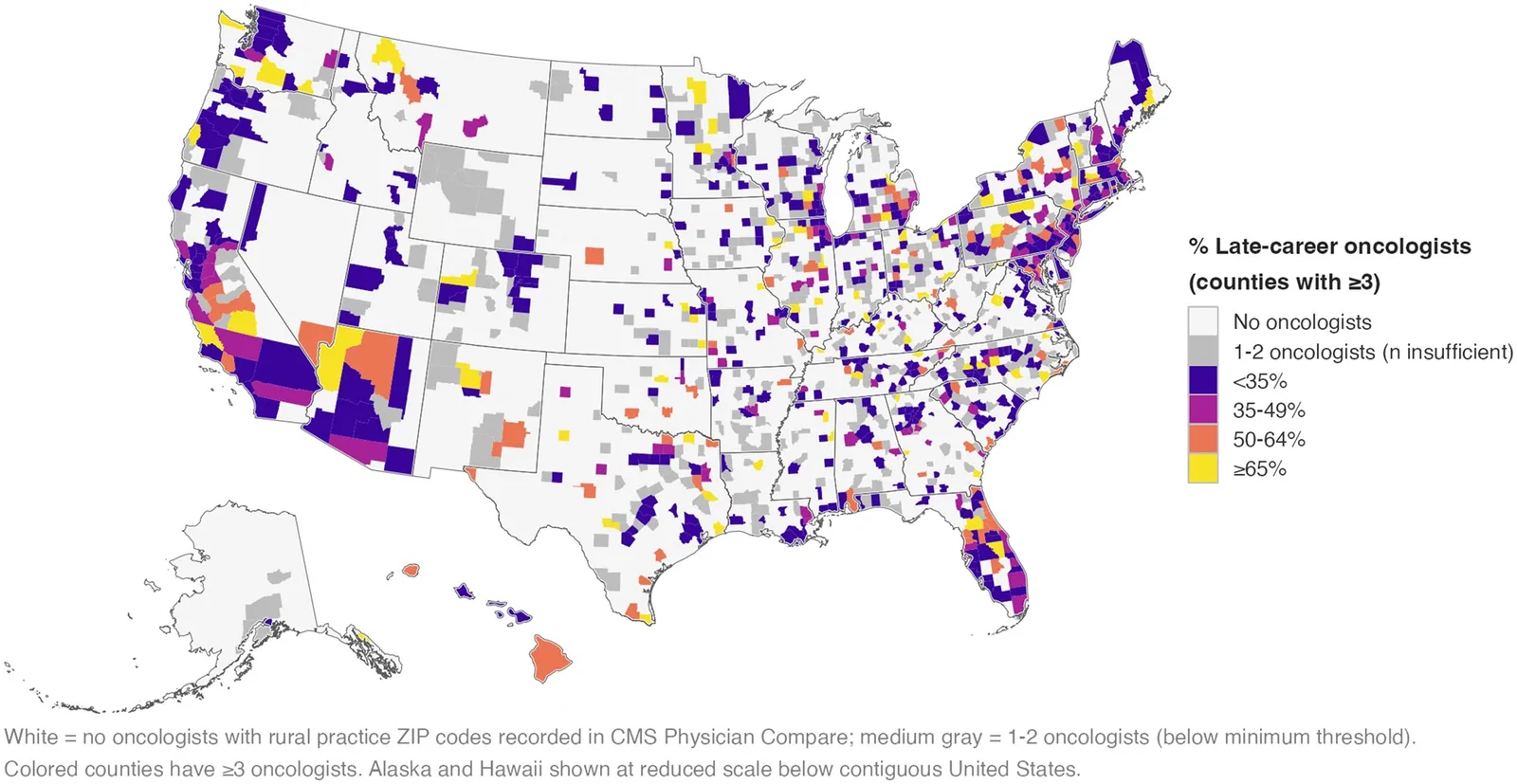
Percentage of late-career US oncology physicians by county, 2025. County-level data on oncologist career stage were derived from the CMS Physician Compare Database. Rural designation was applied using 2010 USDA Rural-Urban Commuting Area codes (4-10 = rural). Late career was defined as ≥25 years since graduation (adjusted from 6-year training). County-level spatial boundaries were obtained from US Census Bureau TIGER/Line Cartographic Boundary Files. Counties with fewer than 3 rural oncologists were excluded from the percentage calculation to reduce small-denominator instability.

County-level geographic distribution of late-career oncologist concentration among rural counties with ≥3 oncologists is presented in Figure 3; the majority of threshold-meeting counties (61.8%) had late-career shares below 35%, though 77 counties (11.1%) had late-career shares of 65% or greater, distributed without strong regional clustering. Among rural oncologists, practice was concentrated in micropolitan communities (RUCA 4-6), accounting for 76.2% in 2025 versus 73.4% in 2014. Meanwhile, rural practice in small town and isolated areas (RUCA 7-10) fell from 26.6% to 23.8%, and rural oncologist count in the most remote communities (RUCA 10) declined from 77 to 48 oncologists. The proportional decline was found to be directionally robust to stricter definitions of rurality, as stratification by RUCA 7-10 (τ = −0.55, *P *= .01) and exclusion of secondary metropolitan commuting flows (τ = −0.70, *P *= .001) both preserved statistically significant decreasing trends.

## Discussion

The proportion of oncologists practicing in rural areas declined significantly over the 11-year study period, from 11.1% in 2014 to 8.0% in 2025, even as the total oncology workforce increased by 26%. The absolute number of rural oncologists remained essentially flat over this period, indicating that growth in the field has accrued almost entirely in urban practice settings. These findings extend a body of cross-sectional literature documenting disproportionate urban concentration of oncologists within the United States.^9^^,^^14^^,^^15^^,^^23–27^ Prior workforce analyses across medical specialties, including gastroenterology,^19^ cardiology,^28^ and general surgery,^29^ have documented stable or only modestly changing rural physician proportions over comparable time horizons, suggesting that the significant decline observed in this study is not a generic feature of specialty workforce growth, but may reflect dynamics specific to oncology, such as geographic concentration of cancer center infrastructure,^24^ systemic therapy administration provider requirements,^30^ and differential fellowship training pipelines.^31^

A recent analysis by Kirkwood et al.^9^ documented declining oncologist density per capita and county-level variation in late-career concentration using Medicare billing data from 3 time points between 2014 to 2024, finding that most states with declining per-capita density had limited rural workforce coverage. The present study extends this work by characterizing annual trends in the rural proportion of the oncology workforce across twelve consecutive cross-sections, applying a validated ZIP code-level RUCA classification, and identifying physician and practice characteristics independently associated with rural practice through multivariable logistic regression. Collectively, these findings suggest that the rural-urban disparity in oncology is not static, but actively widening, and that this pattern is unlikely to self-correct given the workforce characteristics described below. Notably, our analysis demonstrates that rural oncologist supply declined in absolute per-capita terms even as urban supply grew, extending Kirkwood et al. by quantifying the widening rural-urban density gap on an annual basis, as well as by identifying the physician- and practice-level correlates of rural practice through multivariable regression.

Later career stage strongly predicted rural practice, with both mid-career (aOR 1.21) and late-career (aOR 1.58) oncologists more likely to practice rurally than early-career oncologists. In conjunction with the observed decline in rural oncologist supply over the study period, this finding raises the concern that the rural oncology workforce is sustained to a greater degree by physicians approaching the end of their careers, as reflected by the strong independent association between late career stage and rural practice, with early-career oncologists underrepresented in rural relative to urban settings and therefore perhaps not adequately poised to replace them.^8^ County-level data reinforce this concern. While most rural counties with sufficient (ie, ≥3) oncologist supply skewed toward younger career stages, 77 counties had late-career shares of 65% or greater, representing national focal points of acute succession risk. Without targeted intervention, eventual retirement of this cohort will translate into further rural attrition,^11^^,^^32^^,^^33^ accelerating the trends observed in our study.

Female sex was associated with modestly decreased odds (aOR 0.86) of rural practice, consistent with prior workforce literature documenting lower rates of rural practice among women across multiple specialties.^34–36^ The structural determinants of this association likely include differential access to spousal employment opportunities, childcare infrastructure, and collegial support networks in rural settings, rather than individual preference.^37–39^ This finding is particularly relevant given the increasing representation of women in the oncology workforce,^40^ and the aggregate impacts of fewer female oncologists entering rural practice are likely to compound unless these structural barriers are addressed.

AMC affiliation demonstrated the largest effect in the model (aOR 0.10), reflecting the near-complete absence of oncologists operating within the academic medical infrastructure from rural practice. Only 2.3% of rural oncologists practiced at confirmed AMCs compared to 24.2% of urban oncologists. Intuitively, this finding is in part structurally determined, as AMCs are by definition geographically concentrated in metropolitan areas.^14^ More consequential is the implication that rural oncology care is delivered almost entirely by community-based physicians operating outside academic infrastructure.^10^ This renders rural cancer care access particularly sensitive to the economics of community oncology practice, including escalating drug acquisition costs, constrained 340B program participation, and reimbursement structures that increasingly favor large integrated health systems over independent community practices.^30^^,^^41^

The group size findings are consistent with this interpretation. Both small (aOR 0.57) and large (aOR 0.40) practice groups were less likely to be rural than medium-sized groups, which appears to serve as the modal practice environment for rural oncologists. The large group association likely reflects the increasing consolidation of oncology into major urban health systems,^42^^,^^43^ a trend contributing to reduced geographic reach of the oncology workforce following closures and consolidation of smaller practices.^16^ The small group association may reflect the financial fragility of small oncology practices, where the cost structure of systemic therapy administration creates economies of scale that these practices cannot sustain.^44^ Medium-sized practices (ie, 10-99 providers) appear best positioned to maintain rural viability.^10^ As group size in this dataset indexes at billing organization scale rather than by local site staffing, the medium group predominance among rural oncologists likely represents affiliation with regional multi-site networks that maintain a presence in small metropolitan (ie, micropolitan) hubs while extending coverage to surrounding rural areas. The field-wide movement of oncologists into larger practice organizations is structurally difficult to sustain in population-sparse rural markets,^45^ where low patient volumes cannot support large local staffing.^44^ This dynamic is largely specific to oncology given the capital required to administer systemic therapies,^46^ and the concentration of academic and integrated-system infrastructure in metropolitan areas.^24^

Regional variation was consistent with population distribution and prior specialty workforce literature. The Midwest was overrepresented among rural oncologists (aOR 1.62), while the Northeast (aOR 0.61) and West (aOR 0.63) were underrepresented, relative to the South. The coastal underrepresentation likely reflects the higher density of academic and large health system infrastructure in those regions,^47^^,^^48^ as well as higher overall urbanization rates. The Midwest finding may reflect differential training pipeline effects (eg, rural training exposure during residency and fellowship has been associated with subsequent rural practice,^49^ and perhaps more structured rural rotation opportunities exist in Midwestern training programs), though the present dataset does not permit direct evaluation of this hypothesis.

Within oncology subspecialty designations, hematologists were substantially less likely (aOR 0.55), while medical oncologists were moderately more likely (aOR 1.20), to practice rurally than hematology/oncology physicians. This gradient likely reflects the clinical infrastructure requirements of hematologic malignancy management,^50^ including bone marrow biopsy capability, specialized laboratory infrastructure, and access to stem cell transplantation, all of which tend to be concentrated at tertiary care centers.^51^ Medical oncologic management of solid tumors, by contrast, is more amenable to community-based delivery, including infusion center administration of standard regimens.^52^^,^^53^ Rural patients with hematologic malignancies therefore face a compounded access disadvantage, comprising both reduced access to local hematology physicians and disease biology often necessitating referral to high-volume centers regardless of local hematologist supply.

The significant decline of rural oncologist representation over the past decade, occurring against a backdrop of overall workforce growth and rising rural cancer burden,^3^ warrants targeted policy attention. Existing physician workforce recruitment and retention programs (eg, J-1 visa waiver programs, National Health Service Corps loan repayment, Conrad 30 waivers) could be more deliberately targeted toward oncology in rural shortage areas.^32^^,^^54^ Fellowship training programs may consider incorporating structured rural rotation requirements, given evidence that rural training exposure predicts rural practice.^55^ Implementation of telemedicine models for systemic therapy monitoring, symptom management, and survivorship offer an adjunctive strategy to extend the rural reach of oncologists without requiring their physical relocation, though disparities in reliable Internet access among rural populations limits the practicality of this solution.^56^ Our findings reaffirm that community oncology practice is the near-exclusive model for rural cancer care delivery,^57^ and in light of consolidation pressures continuing to reshape the oncology practice landscape,^26^ payers and policymakers must prioritize frameworks (eg, value-based alternative payment models^58^) that ensure the viability of community oncology practice in rural areas. Rural cancer mortality is not inevitable. Physician supply is a modifiable upstream factor associated with the rural–urban disparities in cancer outcomes that continue to widen. National workforce projections reinforce this urgency. The Health Resources and Services Administration projects that national demand for oncologists will continue to outpace supply over the coming decade.^59^ Although its models characterize the aggregate oncology workforce as “adequate,” these projections mask the maldistribution documented in the present analysis, in which rural staffing is demonstrably inadequate relative to the at-risk population.

This study has several limitations. First, the CMS Physician Compare Database relies on PECOS self-report, which may introduce misclassification in specialty designation and practice location. Second, the database captures physicians participating in Medicare, encompassing the large majority of practicing oncologists but potentially excluding a small number of physicians with predominantly younger patient panels. Third, career stage was approximated from graduation year using a uniform 6-year training offset, which does not account for variation in fellowship duration or gaps in training. Fourth, RUCA classification at the ZIP code level may misclassify physicians practicing near rural-urban boundaries. Fifth, multivariable logistic regression analysis identifies associations rather than causal relationships, and the dataset does not capture other key workforce dynamics such as physician workload, patient volume, or career transition patterns over time. Finally, as rurality was assigned from a single primary practice location, urban-based oncologists who deliver intermittent outreach or hub-and-spoke care to rural communities,^60^ a model documented in several states and common in parts of the Midwest,^61^ would be classified as urban. Our estimates therefore capture only where physicians are primarily enrolled rather than the full geographic reach of rural cancer care, likely understating oncologist presence in rural areas. Expansion of such outreach models may represent a more sustainable strategy for extending rural cancer care than relocation-based recruitment, and warrant dedicated study.

## Conclusions

The rural oncology workforce is declining proportionally despite overall growth in the field, driven by urban-concentrated expansion rather than absolute rural attrition. Late career stage strongly positively predicts rural practice, and the underrepresentation of early-career oncologists in rural settings suggests that retirement of the current rural cohort will not be offset by natural workforce replacement. Targeted interventions are needed to prevent further erosion of rural cancer care access. As rural Americans continue to bear a disproportionate burden of cancer incidence and mortality, ensuring an adequate and sustainable oncology workforce in rural communities represents an urgent and actionable public health priority.

## Data Availability

Data available upon reasonable request.
